# Sheaths are diverse and abundant cell surface layers in archaea

**DOI:** 10.1093/ismejo/wrae225

**Published:** 2024-11-05

**Authors:** Sofia Medvedeva, Guillaume Borrel, Simonetta Gribaldo

**Affiliations:** Institut Pasteur, Université Paris Cité, Microbiology Department, Evolutionary Biology of the Microbial Cell, 25 rue du dr Roux, 75015, Paris, France; Institut Pasteur, Université Paris Cité, Microbiology Department, Evolutionary Biology of the Microbial Cell, 25 rue du dr Roux, 75015, Paris, France; Institut Pasteur, Université Paris Cité, Microbiology Department, Evolutionary Biology of the Microbial Cell, 25 rue du dr Roux, 75015, Paris, France

**Keywords:** Sheath, archaea, viruses, methanogens, ANME-1, cell envelope

## Abstract

Prokaryotic cells employ multiple protective layers crucial for defense, structural integrity, and cellular interactions in the environment. Archaea often feature an S-layer, with some species possessing additional and remarkably resistant sheaths. The archaeal sheath has been studied in *Methanothrix* and *Methanospirillum,* revealing a complex structure consisting of amyloid proteins organized into rings. Here, we conducted a comprehensive survey of sheath-forming proteins (SH proteins) across archaeal genomes. Structural modeling reveals a rich diversity of SH proteins, indicating the presence of a sheath in members of the TACK superphylum (*Thermoprotei*), as well as in the methanotrophic ANME-1. SH proteins are present in up to 40 copies per genome and display diverse domain arrangements suggesting multifunctional roles within the sheath, and potential involvement in cell–cell interaction with syntrophic partners. We uncover a complex evolutionary dynamic, indicating active exchange of SH proteins in archaeal communities. We find that viruses infecting sheathed archaea encode a diversity of SH-like proteins and we use them as markers to identify 580 vOTUs potentially associated with sheathed archaea. Structural modeling suggests that viral SH proteins can form complexes with the host SH proteins. We propose a previously unreported egress strategy where the expression of viral SH-like proteins may disrupt the integrity of the host sheath and facilitate viral exit during lysis. Together, our results significantly expand knowledge of the diversity and evolution of the archaeal sheath, which has been largely understudied but might have an important role in shaping microbial communities.

## Introduction

Prokaryotic cells possess multiple protective layers that are crucial for defense against environmental hazards, for maintaining cell shape, for facilitating adhesion to surfaces, and for cell-to-cell interactions [1]. These layers also represent a challenge for entry and egress of viruses, which have developed many ways to cope with them.

Most archaea harbor an S-layer, which is a uniform 2D protein lattice that in some cases is reinforced with additional elements such as polysaccharides, a secondary S-layer, or a tubular sheath [1]. The discovery of sheaths in archaea was made through detailed microscopic studies in *Methanospirillum* (order *Methanomicrobiales*) and *Methanothrix* (order *Methanotrichales*) [2, 3]. This revealed long tubular filaments enclosing multiple cells, separated by plugs which vary significantly between the two species, indicating a distinct architectural approach to cellular organization [4, 5].

The sheath's structure is intricately formed by amyloid proteins organized into hoops which stack sequentially to construct the tubular sheath encasing the cells [6–8]. The remarkable chemical and thermal resistance of the sheath is ensured by cross-beta structures between sheath protein monomers (hereafter referred as SH proteins) and disulfide bonds that interlink the hoops [9, 10]. This structural complexity was further elucidated by the identification of the main SH proteins in *Methanospirillum [**11**]* and *Methanothrix [**12**]* by mass spectrometry (MS/MS). Multiple homologues of *Methanospirillum* SH proteins were found in other *Methanomicrobiales* genomes, whereas *Methanothrix* SH proteins had no homologues in the RefSeq database in 2018 [11]. Finally, a recent study confirmed the originally identified sequence of the main SH protein of *Methanospirillum* by cryogenic electron tomography (cryoET) and have shed light on the molecular organization and assembly of the sheath, providing insights into its biogenesis and the transport of its components across the cell [13].

The presence of sheaths has been documented by microscopy in microbial mats from cold seeps and hydrothermal sediments enriched in ANME-1b [14–16] and in ANME-1a [17–19] archaea, respectively, yet the specific proteins that constitute these sheaths remain unidentified. The limited available microscopy data for uncultured archaea further hints at a broader, yet unrecognized, distribution, and function of sheaths in archaeal communities [20].

Here, we conducted a thorough search for SH proteins in archaeal genomes. We identified multiple SH proteins homologues in members of *Methanotrichales*, *Methanomicrobiales*, *Alkanophagales* (ANME-1), and *Thermofilales*. These SH proteins harbor additional domains, hinting at diverse functions of the sheath. We highlight a complex evolutionary dynamic of archaeal sheaths involving horizontal gene transfers, duplications, and losses of SH proteins. We identified SH-like proteins encoded by multiple (pro) viruses infecting archaea and we propose a lysis mechanism to egress from sheathed hosts. Finally, we use viral SH-like proteins as new markers to identify almost 500 viral species in public databases.

## Materials and methods

### Identification of sheath proteins

We performed an exhaustive search of SH proteins in archaea using previously identified SH proteins of *Methanospirillum hungatei* JF-1 (WP_011449234.1) [11, 12] and *Methanothrix thermoacetophila* PT (/*Methanosaeta thermophila*; ABK14853.1 – discarded later) [12]. First, we identified structural homologs of these proteins using Foldseek [21] in Uniprot50 database [22]. SH protein of *Methanospirillum* (WP_011449234.1) has structural homologs in four taxonomic groups: *Methanotrichales*, *Methanomicrobiales*, *Alkanophagales,* and *Thermofilales*. Foldseek search with SH protein of *Methanosaeta thermoacetophila* (ABK14853.1) produced only a limited number of hits and thus was discarded. Second, we performed a profile search (hmmsearch -E 0.00001) [23] within our custom archaeal database (dereplicated archaeal genomes from GenBank (2023) and archaeal genomes from Nayfach et al. [24]) using an hmm model constructed from hits identified by Foldseek [21]. Hmm profiles were constructed based on protein alignments made using MAFFT with—auto option [25] (v7.453). The iterative profile search was implemented (three iterations)—after the initial hmm search, newly found sequences were realigned with MAFFT and an updated profile was created. After three iterations, no new sequences were found in the database. The full list of genomes used is available in Supplementary Table S1a. The final hmm profiles of SH proteins are available in Supplementary Data 2.

### Phylogenetic analysis

Taxa were selected to cover the phylogenetic diversity of four archaeal orders containing representatives with an observed or predicted sheath layer, *Methanosarcinales*, *Methanomicrobiales*, *Syntropharchaeales,* and *Thermofilales*. The phylogenetic trees are based on a concatenation of sequences of 36 phylogenetic marker proteins from the Phylosift dataset [26] together with sequences of L30 and S4 ribosomal proteins and the A and B subunits of the RNA polymerase. Proteins were retrieved from the selected proteomes with an hmm search (hmmer v3.3.2) [23], aligned with MAFFT (v7.453) [25] with the accuracy-oriented methods, L-INS-i, trimmed with BMGE (v2) with the Blosum30 parameter, and concatenated. For each archaeal order, a maximum likelihood tree was built with IQ-TREE (v2.0.6) [27] using the LG + F + R10 model.

For the tree of SH proteins, 28 representatives of SH proteins with AlphaFold2 predicted structures were aligned using DALI server [28], the alignment was enriched with remaining 168 sequences of SH proteins (Supplementary Table S2) using MAFFT [25] (v7.453) with -add option. The alignment was trimmed to include only core sheath structure (two beta-sheets). A maximum likelihood tree was built with IQ-TREE (v2.0.6) [27] using the Q.pfam + F + R5 model. The alignment is provided in Supplementary Data 3.

### Identification of SH-like proteins in viruses of archaea

SH proteins in archaea possess two key features: a signal peptide, necessary for protein export, and a core amyloid structure. Host sheath proteins without Ig-like domains range from 150 to 350 amino acids in length. We used these characteristics to search for SH proteins in viral genomes. The main rationale behind this approach is to employ sequence-independent methods, as viral SH-like protein sequences may be too divergent from those of the host for direct sequence comparison. Sequences of viruses infecting sheathed archaea were collected from previous studies [29, 30]. Proteins were identified with prodigal (ver 2.6.3 -p meta) [31], and annotated using Prokka (ver 1.14.6) [32] and PHROGs [33] database. We selected proteins with a signal peptide (using SignalP [34] ver 5.0, organism = archaea) and a length ranging from 150 and 350 aa. We predicted the structure of selected proteins using AlphaFold2 [35] and manually assessed the structure in ChimeraX [36]. Using blastp (−evalue 1e-10) additional related viruses were identified in IMG/VR database (ver 4) [37] using sequences of SH-like proteins and MCP of previously identified viruses collected from previous studies [29, 30]. Gene-sharing network was constructed by vConTACT2 [38] with the default parameters.

## Results

### Diversity of sheath proteins in archaea

**Figure 1:**
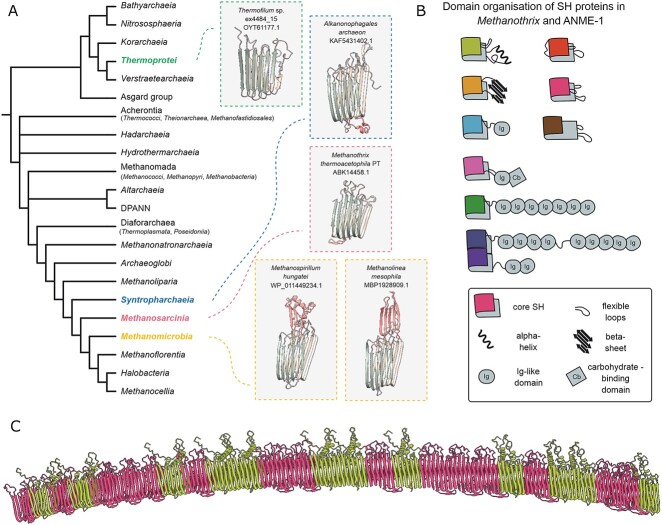
**Diversity of sheath proteins in archaea. A**. Representatives of sheath proteins (SH proteins) in archaea. The structures of SH proteins were obtained with AlphaFold2 after removal of signal peptide. The core amyloid-like sheath structure and additional domains (cap domains) are shown with different colors. **B**. Diversity of cap domains in SH proteins of *Methanothrix*. SH proteins with multiple Ig-like domains (sheath adhesins) are shown in green and dark blue. **C**. AlphaFold2 prediction of the continuous sheath ring structure of *Methanothrix thermoacetophila* PT consisting of two most transcribed SH proteins Mthe_0668 (pink) and Mthe_0667 (green). The colors correspond to panel B.

We conducted a comprehensive search for sheath-forming (SH) proteins in archaeal genomes by leveraging the structural templates of the two previously identified main SH proteins from *M. hungatei* JF-1 (WP_011449234.1, identified by MS/MS and cryoET; [11, 13]) and *M. thermoacetophila* PT (ABK14853.1, identified by MS/MS; [12]). Briefly, we used Foldseek [21] for structural homologue identification, followed by search with specific hmm profiles [23]. We found no structural homologue of SH-proteins in Bacteria, suggesting that sheath structure as described in *Methanothrix*/*Methanospirillum* is likely unique to the archaeal domain. This led us to identify 643 structural homologues of *Methanospirillum* SH protein in 48 representative archaeal genomes, frequently present in multiple copies per genome (Supplementary Tables S1 and S2). These SH proteins are distributed across diverse lineages, namely in all members of the order *Methanotrichales* (*Methanosarcinia*), not only in *Methanoregulaceae* and *Methanospirillacae* families (*Methanomicrobia*) but also in 17% of *Thermofilales* as well as 40% of *Alkanophagales* (ANME-1) (Fig. 1A). These data extend the presence of a sheath in a larger spectrum of archaeal diversity than previously known and support the presence of a sheath on ANME-1 cells suggested by microscopy observations of environmental samples and enrichment cultures [17–19]. In contrast, homologues of the SH protein previously identified in *M. thermoacetophila* PT (ABK14853.1) and proposed to be the main sheath components [12]) are only found in a few *Methanotrichales* strains (Supplementary Table S1c). This limited distribution, as well as absence of dimerization according to AlphaFold2, and absence of structural similarity to the *Methanospirillum* main sheath protein suggest that this protein was thus likely misidentified by MS/MS as the main sheath protein but may instead be a specific and perhaps secondary sheath component.

All identified SH proteins share a core amyloid-like structure of two β-sheets, each formed by six anti-parallel β-strands (Fig. 1A). This core structure is enhanced by diverse “cap” domains (Fig. 1A) that have been shown to face the exterior of the sheath filament and contribute to the sheath's structural integrity and function [13]. In *Methanospirillum*, the cap domain is made of six α helices and one β sheet (Fig. 1A), and it was shown to play a role in connections between individual hoops of the sheath [13]. In *Methanolinea*—another member of *Methanomicrobiales*—we infer that the cap domain is made of a 5-strand β sheet, indicating cap diversity even among closely related archaea. Analysis of *Methanothrix* and ANME-1 SH proteins identified a large structural variety, with a wide range of cap domains (e.g., no cap domain, single α helix, two β sheets, Immunoglobulin-like (Ig-like) domain, Ig-like, and carbohydrate-binding domain, or several Ig-like domains connected by flexible loops) (Fig. 1B, Supplementary Fig. 1). Moreover, 63 proteins with repeated SH core structures (2–3 consecutive domains) were found (Supplementary Table S1b). Despite the structural variety of cap domains, we could not associate any domain organizations to specific lineages, as most architectures of SH proteins are present across *Methanothrix* strains, often in multiple copies (Supplementary Fig. 1).

AlphaFold2 prediction suggests that the individual rings of the sheath could be made of multiple SH proteins with different cap domains forming a continuous cross-β structure (Fig. 1C). This hypothesis is supported by analysis of available transcriptomic data from *M. thermoacetophila* PT [39] and *M. hungatei* JF-1 (BioProject PRJNA263077). In fact, three SH proteins with distinct cap domains are the most highly transcribed in *Methanothrix* (Mthe_0668, Mthe_0667, and Mthe_0625, ratio 100:67:29) and two SH proteins in *Methanospirillum* (Mhun_2271 and Mhun_1947, ratio 100:13) (Supplementary Table S1d). These proteins are likely the main components of the sheath in these two archaea and may assemble in corresponding proportions in the structure (Fig. 1C). The remaining SH proteins show very low transcription levels (Supplementary Fig. 2). They may be expressed in specific conditions or perhaps needed in less abundance. For example, SH proteins with small cap domains might connect individual hoops or support the plugs, similarly to what shown in *Methanospirillum* [13]. SH proteins with a long chain of Ig-like domains could form sheaths with adhesion properties, replacing classical membrane-anchored adhesion proteins. Finally, unlike other homologues, the SH proteins in members of *Thermoprotei* do not have any additional domains (Fig. 1A). We found SH proteins in several uncultured and unclassified MAGs in *Thermofilales*. The cultured relatives *Thermofilum* spp*.* make long multicellular filaments, but do not have SH homologues and a sheath has not been highlighted by microscopy [40, 41]. Together, these results show variability in cap domain structures, suggesting specialized roles for each SH variant in the sheath assembly and highlighting the complexity of sheath structures in archaea.

**Figure 2:**
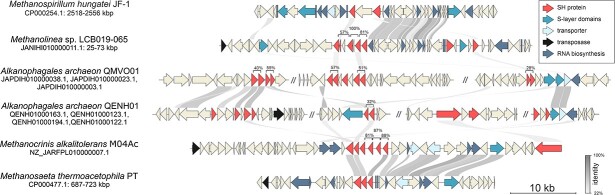
**Genomic context of SH-proteins.** Six representative genomes of sheath-containing archaea are presented. SH-proteins of *Alkanophagales* archaeon QMVO01 and QENH01 are encoded in three and four contigs respectively. The ends of contigs are shown by // symbol. The protein identity (calculated by blastp) between neighboring SH-proteins is shown above the brackets. The protein identity between different genomes is shown by grey links.

The protein composition of the plug and how plug formation is coordinated with the cell division are still poorly understood [42]. Moreover, it has remained unclear how new hoops are inserted into the sheath and what are other proteins participating in the sheath assembly. Unfortunately, the genomic context of SH proteins (Fig. 2) provides no suggestion for proteins involved in sheath assembly and plug formation. The SH protein-encoding genes have a tendency towards duplication, likely aimed at enhancing sheath production. As a result, SH protein-encoding genes are in clusters, with up to 100% protein identity among neighboring copies, suggesting that these gene duplications are relatively recent events. *Methanothricales* and *Methanomicrobiales* genomes contain a single cluster of SH protein-encoding genes, whereas *Alkanophagales* (ANME-1) encode up to four clusters (Fig. 2). However, aside from the SH-encoding genes themselves, no conserved genomic context was observed. Nevertheless, SH genes are in the vicinity of genes coding for core housekeeping functions, such as tRNA and rRNA biosynthesis, generic transporters, and genes coding proteins with S-layer-like domain (Fig. 2), indicating a key cellular function of the sheath. Additionally, we used comparative genomics to identify plug proteins and sheath chaperones. Unfortunately, due to lack of conserved genomic context and high divergence of ANME-1, *Methanotrichales*, and *Methanomicrobiales* archaea, we were unable to associate additional proteins with sheath and plug formation. Nevertheless, we found thiol-exchange protein (DbsB) and its membrane partner (DbsD) [43] in sheath-containing *Methanomicrobiales* and *Methanotrichales* (Supplementary Table S1e). Disulfide bonds have been shown to be important for the sheath integrity providing connections between individual hoops [13]. DbsB and DsbD are present in other archaeal lineages such as *Halobacteria* and thus are not specific to the formation of the sheath.

### Evolutionary dynamics of the sheath

**Figure 3:**
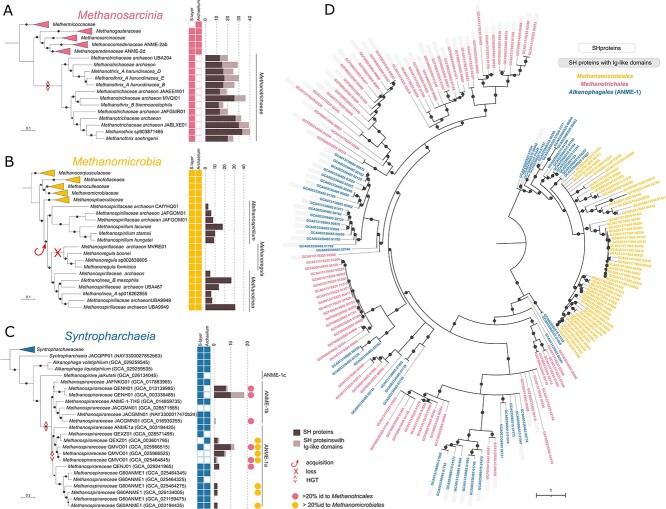
**Evolutionary history of sheath in archaea.** Distribution of SH proteins in *Methanosarcinia* (**A**), *Methanomicrobia* (**B**) and *Syntropharchaeia* (**C**). The presence/absence of S-layer and archaellum is shown on the right of the trees. The bar plot shows the number of copies of SH proteins found in the genome without additional domains (darker shade) or with Ig-like domains (lighter shade). The tree of *Syntropharchaeia* (**C**) contains information about closest homologues of SH proteins from *Methanotrichales* (pink dot) or *Methanomicrobia* (yellow dot). **D.** Phylogenetic tree of SH core sequence based on structural alignments of core SH domain generated by DALI server. The tree is rooted at midpoint. The bootstrap value >90 is indicated with the dot. The branches with support <70 are collapsed. The color of the leaf font corresponds to panels **A**, **B**, and **C**: *Methanotrichales*, *Methanomicrobiales*, *Syntropharchaeia*. The darker background of the leaf represents SH proteins with Ig-like domains.

Given the patchy distribution of sheaths in archaea, we sought to understand their evolutionary history by mapping the occurrence of SH proteins on the reference trees of the three groups of archaea where they are found (Fig. 3). All *Methanotrichales* members possess multiple SH proteins, with each genome containing up to 40 copies (Fig. 3A). Moreover, they all have a subset of their SH proteins extended with Ig-like domains, likely used for adhesion. In the *Methanomicrobiales*, SH proteins are observed in all members of the *Methanospirillum* and *Methanolinea* but they are absent in the closely related *Methanoregula* (Fig. 3B) [44, 45]. This agrees with microscopy observations of *Methanoregula boonei*, which did not show the presence of sheaths (Supplementary Fig. 3) [46, 47]. Each genome harbors between 3 to 31 SH protein copies, yet none with Ig-like domains. Finally, SH proteins are patchily but widely distributed in *Alkanophagales* (ANME-1) archaea (Fig. 3C), supporting the likely presence of a sheath in this lineage beyond previous microscopy evidence of environmental samples and co-cultures with syntrophic bacteria (Supplementary Fig. 3) [14–19]. Specifically, we found SH homologues in two main ANME clades, ANME-1b (*cand.* Genus QENH01) identified in cold seeps throughout the Atlantic and Pacific oceans [16], and in ANME-1a (*cand.* Genera QEXZ01, QMVO01) discovered in hydrothermal sediments [30, 48]. These genomes can harbor between 1 to 18 copies of SH proteins, occasionally featuring additional domains (Supplementary Table S1a). Intriguingly, some SH proteins in ANME-1 show up to 56% sequence similarity with those found in *Methanotrichales* (pink dots, Fig. 3C) and *Methanospirillum* (yellow dots, Fig. 3C), suggesting horizontal gene transfers among these archaea.

To elucidate the evolutionary patterns leading to SH protein distribution, we performed phylogenetic analysis. Given the low sequence similarity between SH proteins of different archaeal groups we used structure-based alignment of the core SH protein domain for phylogeny reconstruction (Fig. 3D). The resulting tree shows a clear division between *Methanomicrobiales* (in yellow) and *Methanotrichales* (in pink) archaea. Sequences of *Methanomicrobiales* SH proteins are all clustered together and nested within ANME-1a sequences. In contrast, *Methanotrichales* SH proteins are divided into several supported non-monophyletic clades, corresponding to different cap domains arrangements, and intermixed with ANME-1 sequences (Fig. 3D). From this phylogeny, we infer that *Methanotrichales* and ANME-1 exchanged at least five types of SH proteins, encompassing variants with and without specific functional domains. The common ancestor of *Methanospirillum* and *Methanolinea* likely acquired SH proteins, specifically those lacking extra Ig-like domains, from ANME-1, which was followed by a loss of SH proteins in *Methanoregula*. Sheathless *Methanoregula* members have been observed to be dimorphic (thin rods and irregular cocci), and the coccoid cells were suggested to be the product of asymmetric cell division of rod-shaped cells [46, 47]. Asymmetric cell division is rare in archaea, but was observed in other sheathed archaea (*Methanothrix*), where it produces an empty space in the chain of cells, which becomes a breaking point of the sheathed filament [49]. Given that *Methanoregula* likely lost their sheath, asymmetric cell division in this family might be the remnant of an ancestral mechanism for controlling the length of the sheathed filament.

We questioned how the sheath may exclude or add to other important components of the cell envelope: the S-layer and the archaellum (Fig. 3A, B, and C). In most cases, the sheathed archaea also have proteins of the S-layer, similarly to their closest sheathless relatives, suggesting no specific conflict between these two protein layers. Consistently, prior electron microscopy has shown the presence of a S-layer beneath the sheath in *Methanospirillum* [2]. In *Methanotrichales*, no S-layer was reported from previous electron microscopy observation but a thin granular layer on the membrane surface [50]. This layer might be composed of the S-layer protein identified in *M. thermoacetophila* (*M. thermophila*), whose gene is the second most expressed in the cell and is located next to the most expressed SH protein gene (Supplementary Fig. 2, Fig. 2). The situation is more contrasted for the archaellum. Indeed, although the archaellum is present across all *Methanomicrobiales* archaea (Fig. 3B), it is specifically absent from all *Methanotrichales* representatives within the *Methanosarcinia* (Fig. 3A). This suggests that this system was lost in link with the acquisition of the sheath. In other *Methanosarcinia*, the archaellum was proposed to be involved in adhesion rather than motility [51]. It is thus possible that Ig-like domain extensions present on several copies of the SH proteins in *Methanotrichales* may replace this adhesive role. Similarly, *Syntropharchaeia* that have SH proteins with Ig-like domain extension also tend to lack the archaellum even though the archaellum is mostly present in other members of this class (Fig. 3C). These results reveal the complex evolutionary history of SH proteins, indicating a dynamic exchange and evolution of SH proteins within these archaeal communities.

**Figure 4:**
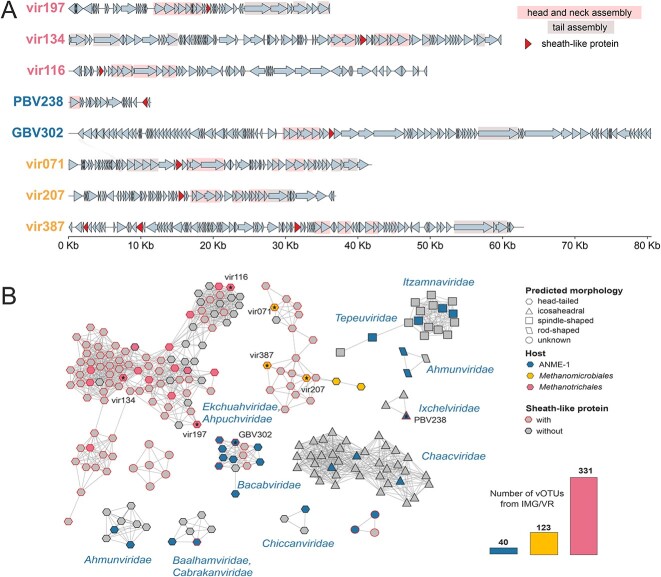
**SH-like proteins in viruses associated with sheathed archaea. A.** Representative genomes of previously reported viruses associated with the sheathed archaea (*Methanotrichales*—pink, *Methanomicrobiales*—yellow, *Syntropharchaeia*—blue fonts). Proteins involved in head and neck assembly are shown with pink background, proteins involved in tail assembly are shown with grey background. The SH-like protein are highlighted. **B**. A gene-sharing network of viruses associated with sheathed archaea (vConTACT2). Each node corresponds to the high-quality vOTU. The predicted morphology of the virus is shown by a node shape. Colored nodes represent reference viruses, collected from previous studies and their respective hosts (*Methanotrichales*, *Methanomicrobiales*, *Syntropharchaeia*). Stars inside the node indicate viruses used in panel a of this figure. Viruses containing SH-like protein are showed with a red outline. The total number of vOTUs which encode SH-like protein from IMG/VR database which is shown on the barplot (*Methanotrichales*, *Methanomicrobiales*, *Syntropharchaeia*).

**Figure 5:**
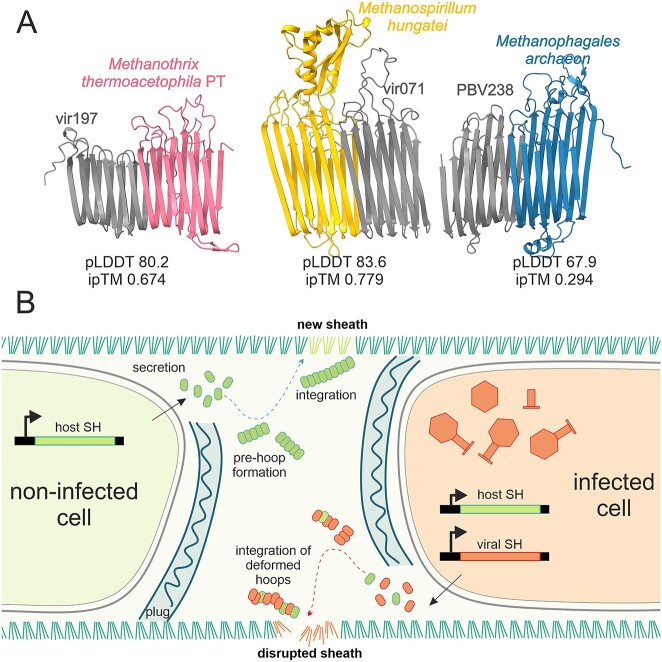
**A proposed model of viral egress mechanism using SH-like proteins. A.** AlphaFold2 prediction of interaction between viral SH-like proteins (vir197, vir071, and PBV238) and corresponding host SH proteins (*Methanotrichales*, *Methanomicrobiales*, *Syntropharchaeia*). pLDDT, predicted local distance difference test (measurement of model confidence); ipTM, interface predicted template modelling score (measurement of interface accuracy). **B**. A proposed model of viral egress mechanism using SH-like proteins. In normal conditions (non-infected cell, left) the host SH protein is secreted outside of the cell where it is assembled in amyloid-like structures (pre-hoops) which are inserted in the sheath (light green, top). In an infected cell (right) the viral SH-like protein is expressed and secreted outside where it tampers with the assembly of sheath. As a result, deformed hoops are integrated in the sheath which reduce the sheath stability and enable viral egress.

### An egress strategy of viruses infecting sheathed archaea

Viruses of archaea employ sophisticated mechanisms to breach the cell walls of their hosts [52]. Bacteriophage-like archaeal viruses with head-tailed morphology produce enzymes (endolysins) that cause cell lysis during viral egress [29, 53]. Rod-shaped and icosahedral viruses employ virus-encoded pyramidal structures (VAPs) that grow and protrude through the S-layer, eventually forming openings for virion exit [52]. Spindle-shaped and pleomorphic viruses are released through budding [54]. Similarly, viruses of sheath-coated archaea must therefore be presented with the challenge of degrading the additional cell wall layer—remarkably resistant amyloid-like structure of the sheath that stays intact even after cell death [15]. This may require specific mechanisms of egress, such as virus-encoded proteases capable of amyloid degradation, or formation of structures disrupting the integrity of the sheath. Recent studies have identified morphologically diverse viruses associated with sheathed archaea [29, 30]. Specifically, these studies identified 22 (pro)viruses with head-tailed morphology associated with *Methanospirillum*, *Methanolinea,* and *Methanotrichales* [29] and 28 from ANME-1 archaea [30] (Supplementary Table S1f).

To identify potential egress-related genes, we re-annotated the genomes of these (pro)viruses and modeled (AlphaFold2 [35]) 87 viral proteins containing signal peptides. Many of these (pro) viral genomes encode proteins with an amyloid-like structure resembling SH proteins (Fig. 4A), 13/16 in *Methanotrichales*, 3/6 in *Methanospirillum* and *Methanolinea* viruses (Supplementary Table S1f). For ANME-1 archaea, only and 7/28 (pro)viruses with head-tailed and icosahedral morphology harbored SH-like proteins (Supplementary Table S1f, Fig. 4B), reflecting the patchy distribution of sheath in ANME-1. Additionally, we searched the IMGVR database for viruses of sheathed archaea using SH-like proteins and major capsid proteins (MCPs) of 50 previously identified (pro)viruses. We identified 580 vOTUs potentially associated with sheathed archaea, including 175 high quality vOTUs (Supplementary Table S1g, Fig. 4B). We found that 75% of *Methanothrix*, 86% of *Methanospirillum/Methanolinea,* and 13.5% of ANME-1 viruses encode SH-like proteins (Fig. 4B), while the remaining were found solely based on the MCP similarity. Given these results, we reasoned that SH proteins might be widely distributed in viruses infecting sheathed hosts and therefore they may be used as additional markers to identify new viruses associated with these archaea.

Virus-encoded SH proteins are 159–288 AA in length and, in most cases, do not carry any cap domains. Despite maintaining the amyloid-like structure, the sequences of SH-like viral proteins are generally highly divergent (Supplementary Data 1). In some viruses (Supplementary Table S1f) the SH-like proteins are 75%–94% identical to the host SH proteins, suggesting recent acquisition of viral SH-like proteins from the host. Alphafold2 predicted a well-supported interaction between virus-like SH proteins and between viral and host SH proteins in all groups of sheathed archaea (Fig. 5A). This potential interaction, together with the presence of signal peptides for secretion, suggests that viral SH proteins may be integrated into the host sheath structure.

From these results, we propose a model of viral egress in sheathed archaea (Fig. 5B). We hypothesize that, in non-infected cells (Fig. 5B, left) the host SH protein is secreted outside of the cell where it is assembled in amyloid-like structures (pre-hoops) which are inserted in the sheath [13]. Conversely, in infected cells (Fig. 5B, right) the viral SH-like protein is expressed and secreted outside where it tampers with the assembly of sheath. Divergent viral SH-proteins may decrease the stability of the sheath or, alternatively, they may disrupt sheath assembly, competing with host SH monomers. As a result, deformed pre-hoops are integrated in the sheath which reduces its stability, enabling viral egress. The acquisition and repurposing of SH-like proteins from the host can be considered as another example of “guns for hire” strategy of viral adaptation [55], where a component of the host defense system (the sheath) is recruited by viruses to facilitate the host lysis.

Other hypotheses may be put forward for the role of viral SH-like proteins. By interacting with the host SH proteins, viral SH-like proteins may direct viral particles or unknown factors involved in the lysis process to the host cell surface. Alternatively, viral SH-like proteins might compete with the host SH proteins for the sheath assembly machinery, slowing down cell growth. Finally, viral SH-like proteins may hijack the host's sheath assembly machinery to assist in viral particle assembly and not be directly involved in lysis. Additional experimental work is needed to distinguish between these potential mechanisms. Together, these results show that SH proteins can be used as additional markers to identify viruses associated with sheathed archaea and suggest that their employment for egress might be a universal adaptation of these viruses. In addition, viruses may have also participated in horizontal gene transfer of SH proteins in archaea.

## Discussion

Archaea exhibit a remarkable diversity in their envelopes, which are involved in adaptation to a wide range of environments and protection against viruses. However, archaeal envelopes remain poorly characterized [1]. In this work we used available genomic data to predict the presence of a sheath in three distant lineages of Methanotecta. We uncovered a large diversity of sheath-forming proteins and explored the evolutionary history of the sheath in archaea. Finally, we found that viruses of sheathed archaea encode sheath-like proteins and propose an egress mechanism from sheathed hosts.

It is likely that sheaths are more widespread in archaea than currently recognized, but high sequence diversity of the SH proteins makes them difficult to identify. Sheaths may be universally present in all members of the order (as in *Methanotrichales*), restricted to a several genera within an order (as in *Methanospirillum*, *Methanolinea*), or found only in a few strains (as in ANME-1, *Thermofilales*). Currently, many proposed taxa of archaea lack a cultured member and are identified solely through metagenomic studies. To confirm the presence of sheaths in these lineages, microscopy studies of environmental samples combined with metatranscriptomic analysis will be necessary.

The variability in the cap domains of SH proteins, as identified across different archaeal lineages, indicates a specialization of sheath structures that could be tailored to specific environmental or physiological needs, serving as platforms for attaching surface functions. The most widespread modification of SH proteins with Ig-like domains (sheath adhesins) observed in *Methanotrichales* and ANME-1 might be a special adaptation to the environments where these archaea thrive or a response to the presence of syntrophic partners [39, 56–58] or parasites [58]. Intriguingly, ANME-1 with its syntrophic partner HotSeep-1 form dense aggregates and bacteria were even pictured inside a sheath of ANME-1 [17, 59]. The syntrophic partners of *Methanotrichales* and ANME-1 have been shown to participate in DIET (direct interspecies electron transfer) using nanowires [18, 39, 56]. Nanowires are electrically conductive filaments produced by syntrophic bacteria (*Geobacter*, sulfate-reducing bacteria HotSeep-1) and connecting bacteria with the sheath of the archaea. However, the conductivity properties of the sheaths are yet to be confirmed.

Among other functions, the sheath likely provides defense against viruses, shielding the cell surface from viral absorption and preventing the delivery of viral DNA into the host cell. In our analysis we identified only viruses of head-tailed and icosahedral tailless morphology that are associated with sheathed archaea. These viruses are likely to inject the genome into the host cell, in a similar fashion as bacteriophages [60]. Icosahedral and head-tailed viruses of bacteria are able to bridge the distance to the host cytoplasm through the peptidoglycan layer using diverse encapsulated viral proteins [61]. A similar mechanism could be responsible for the delivery of viral DNA through the archaeal sheath.

Our findings suggest that the use of SH proteins for viral egress is a common adaptation among viruses infecting sheathed archaea. The ability of these proteins to interact with and potentially destabilize the host sheath underscores their role in facilitating viral exit. Additionally, the presence of SH proteins in a substantial proportion of viruses associated with sheathed archaea indicates that these proteins can serve as reliable markers for identifying new viruses infecting sheath-coated archaeal hosts.

Understanding the diversity and function of archaeal envelopes, particularly sheaths, enhances our knowledge of archaeal biology and their ecological roles. Characterizing sheath structures in archaea through advanced genomic, transcriptomic, and proteomic techniques will provide a more comprehensive understanding of sheath diversity and function. Finally, further exploring the evolutionary dynamics of SH proteins and the involvement of viruses in this process could reveal their impact on archaeal diversity.

## Supplementary Material

Supplementary_Figures_wrae225

Supplementary_tables_wrae225

Supplementary_Data_wrae225

Supplementary-legends_wrae225

## Data Availability

All data generated or analyzed during this study are included in this published article and its supplementary information files (Supplementary Tables S1–7, Supplementary Data 1–3).
