# Supplementary material for: Sheaths are diverse and abundant cell surface layers in archaea
Source: ISME J. 2024 Nov 5;18(1):wrae225. doi: 10.1093/ismejo/wrae225 (PMC11576556; doi:10.1093/ismejo/wrae225)
Supplement: Supplementary_Figures_wrae225 [file supplementary_figures_wrae225.pdf]

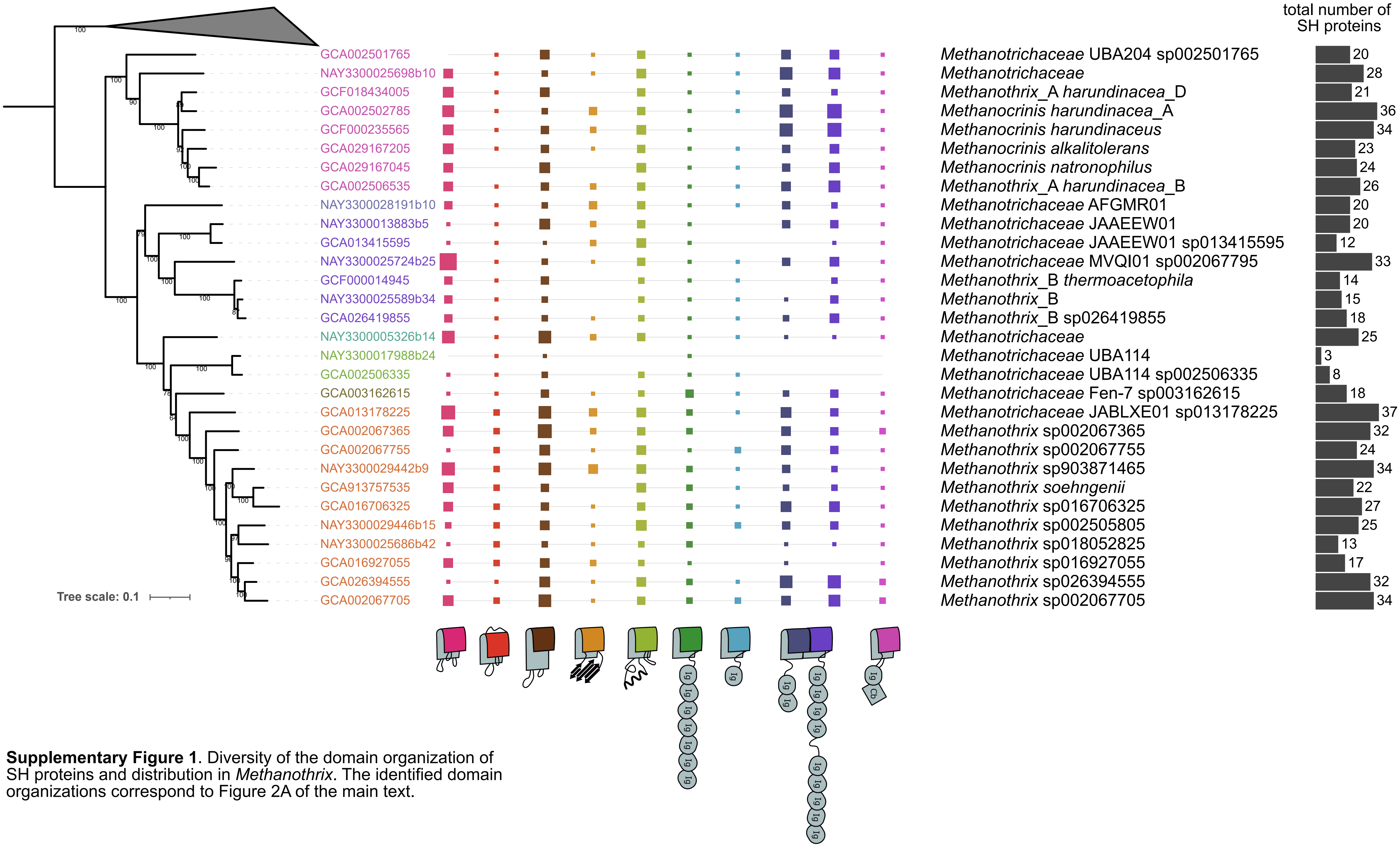

Supplementary Figure 1. Diversity of the domain organization of SH proteins and distribution in *Methanothrix*. The identified domain organizations correspond to Figure 2A of the main text.

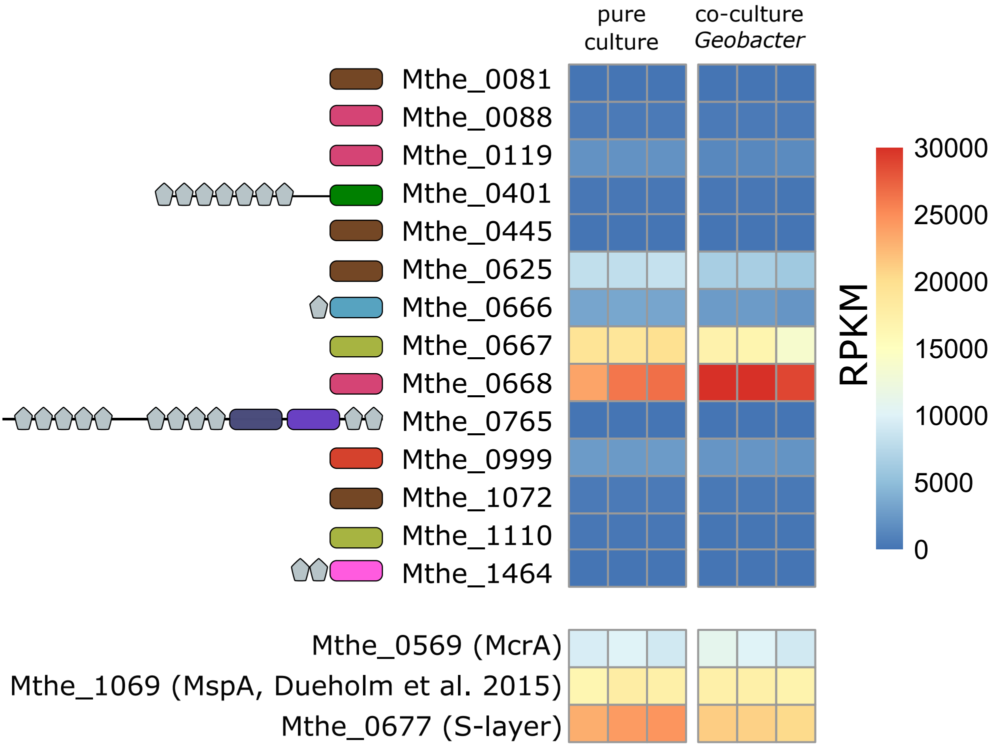

Supplementary Figure 2.

Transcriptomic analysis of SH proteins from *Methanothrix thermoacetophila* PT.
Raw data analyzed from MBio 2023; 14: e0036023.
The heatmap shows transcription level of SH-like proteins in six samples – 3 replicates of *Methanothrix thermoacetophila* PT pure culture and 3 replicated of co-culture with *Geobacter*.
The domain organization of SH-like proteins is shown on the left. The colors correspond to Figure 1B.

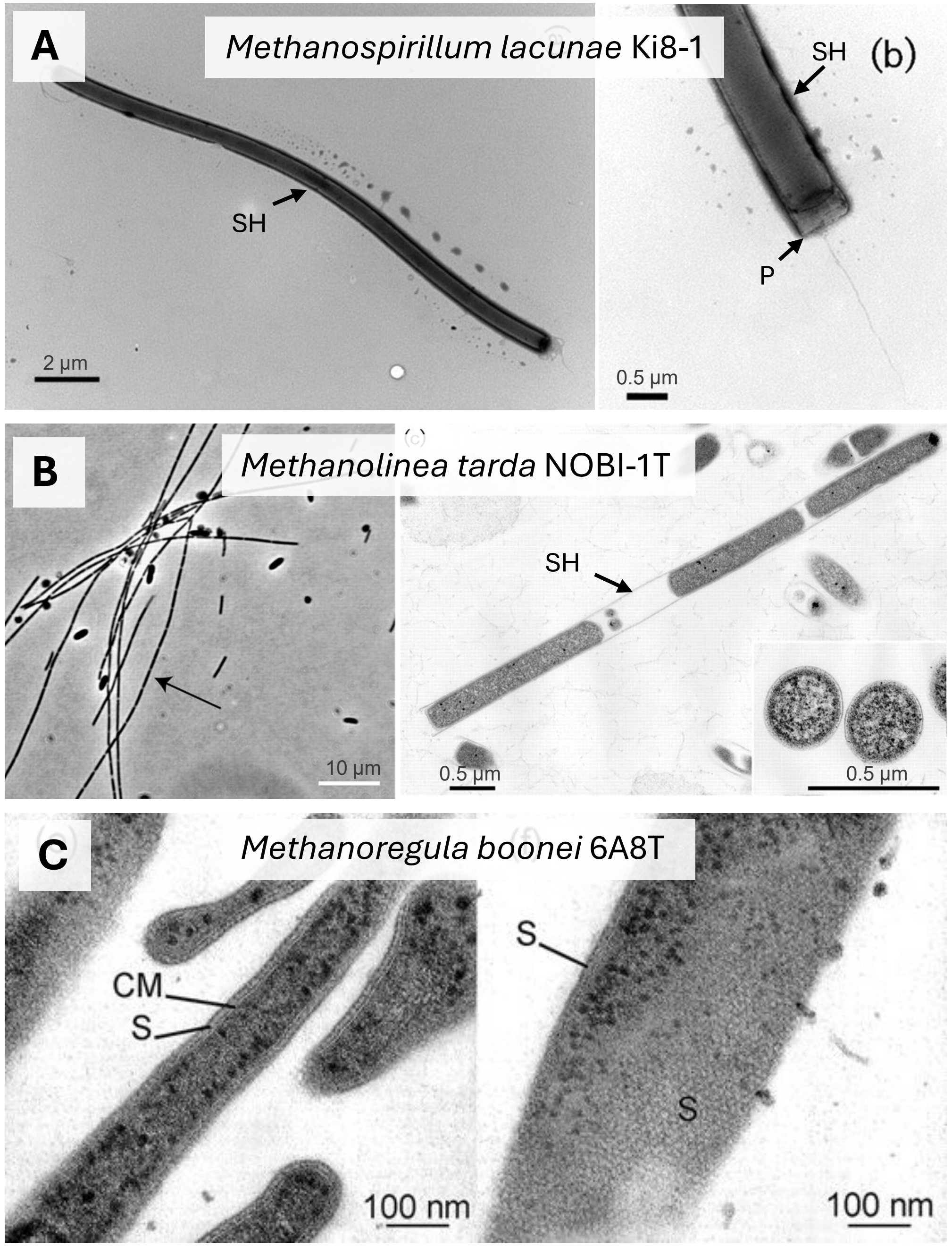

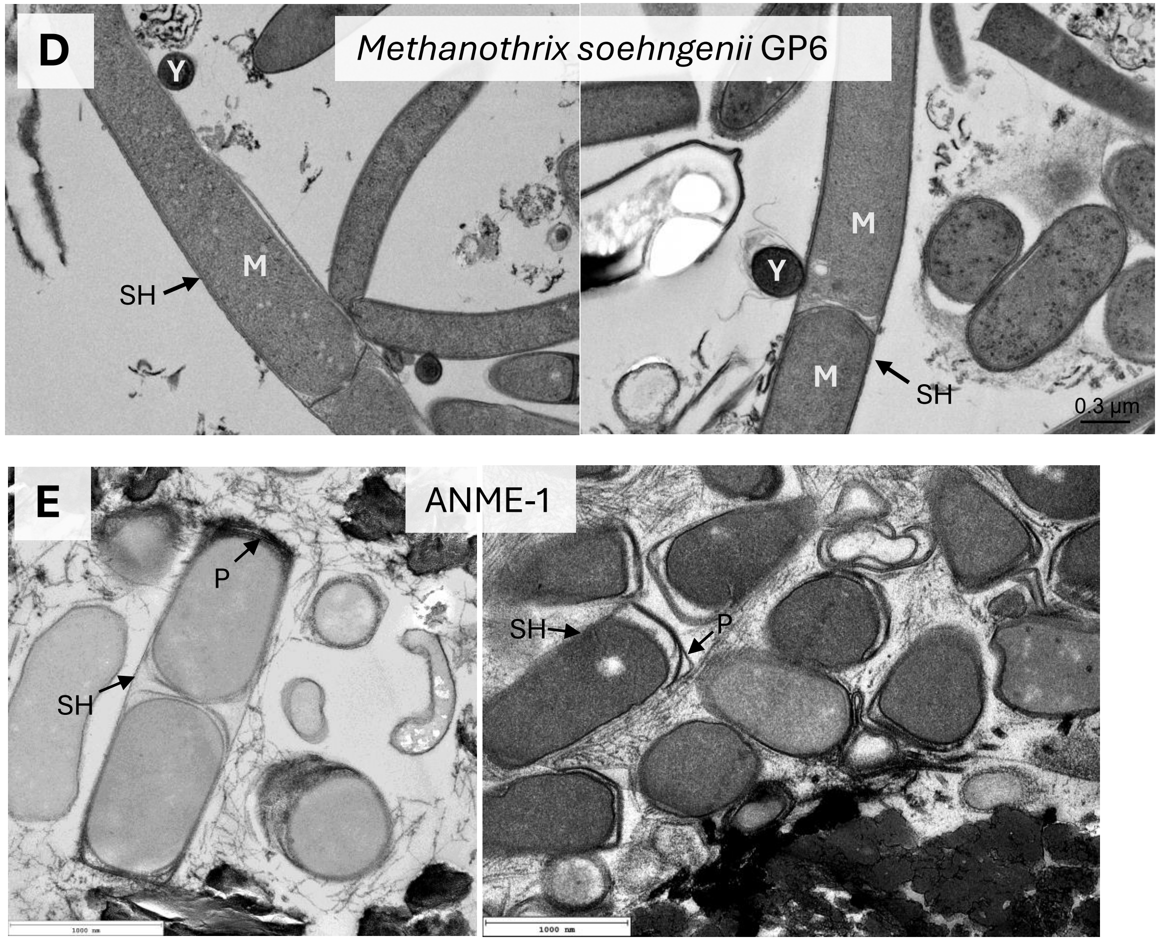

Supplementary Figure 3. Micrographs of sheath-bearing archaea. Arrows indicate SH-sheath, P- plug.

A. Transmission electron micrographs of cells of *Methanospirillum lacunae* strain Ki8-1 with sheath. Reproduced with permission from **Int J Syst Evol Microbiol 60, 2563 (2010);**

B. Micrographs of *Methanolinea tarda* strain NOBI-1T with sheath. Reproduced with permission from **Int J Syst Evol Microbiol 58, 294 (2008);**

C. Thin sections electron micrographs of *Methanoregula boonei* 6A8T without sheath. CM - cytoplasmic membrane, S **–** S-layer. Reproduced with permission from **Int J Syst Evol Microbiol 61, 45 (2011)**

D. Electron micrographs of *Methanothrix* (A) in association with *Ca*. Yanofskyibacterium cells (B), courtesy of Dr. Takashi Narihiro and Dr. Kyohei Kuroda, reproduced with permission from **mBio 2022 Oct 26;13(5):e0171122** (left) and **mBio 2024 Mar 13;15(3):e0310223** (right);

E. Electron micrographs of ANME-1 in association with and bacterial cells (SRB HotSeep-1). Courtesy of Dr. Dietmar Riedel and Dr. Gunter Wegener, reproduced with permission from **Nature. 2015 Oct 22;526(7574):587-90**.
