# Supplementary material for: Sheaths are diverse and abundant cell surface layers in archaea
Source: ISME J. 2024 Nov 5;18(1):wrae225. doi: 10.1093/ismejo/wrae225 (PMC11576556; doi:10.1093/ismejo/wrae225)
Supplement: Supplementary-legends_wrae225 [file supplementary-legends_wrae225.docx]

**Supplementary Figure 1. Diversity of the domain organization of SH proteins and distribution in *Methanothrix*.** The domain organizations and colors correspond to Figure 1B of the main text.

**Supplementary Figure 2. Transcriptomic analysis of SH proteins from *Methanothrix thermoacetophila* PT.** The heatmap shows transcription level of SH-like proteins in six samples – 3 replicates of *Methanothrix* *thermoacetophila* PT pure culture and 3 replicated of co-culture with *Geobacter*. The domain organization of SH-like proteins is shown on the left. The colors correspond to Figure 1B.

**Supplementary Figure 3**. Micrographs of sheath-bearing archaea. Arrows indicate SH-sheath, P- plug.

**A.** Transmission electron micrographs of cells of *Methanospirillum lacunae* strain Ki8-1 with sheath. Reproduced with permission from **Int J Syst Evol Microbiol 60, 2563 (2010);**

**B.** Micrographs of *Methanolinea* *tarda* strain NOBI-1T with sheath. Reproduced with permission from **Int J Syst Evol Microbiol 58, 294 (2008);**

**C.** Thin sections electron micrographs of *Methanoregula boonei* 6A8T without sheath. CM - cytoplasmic membrane, S – S-layer. Reproduced with permission from **Int J Syst Evol Microbiol 61, 45 (2011)**

**D.** Electron micrographs of *Methanothrix* (A) in association with *Ca*. Yanofskyibacterium cells (B), courtesy of Dr. Takashi Narihiro and Dr. Kyohei Kuroda, reproduced with permission from mBio 2022 Oct 26;13(5):e0171122 (left) and mBio 2024 Mar 13;15(3):e0310223 (right);

**E.** Electron micrographs of ANME-1 in association with and bacterial cells (SRB HotSeep-1). Courtesy of Dr. Dietmar Riedel and Dr. Gunter Wegener, reproduced with permission from Nature. 2015 Oct 22;526(7574):587-90.

**Supplementary Table 1**.

1. Archaeal genomes used in the study (dereplicated genomes from GenBank (2023) and archaeal MAGs from Nayfach et al.). The number of sheath proteins (SH proteins) with and without Ig-like domains is specified.
2. Sheath proteins identified in archaeal genomes with number of amyloid core domains.
3. Blast result of previously suggested main sheath protein of *Methanothrix thermoacetophila* PT. This protein was later discarded from analysis because it is likely a secondary sheath component.
4. Transcriptomic analysis of *Methanospirillum hungatei* JF-1 and *Methanothrix thermoacetophila* PT. Top 20 most transcribed proteins are shown.
5. Comparative genomics analysis of archaeal genomes with and without sheath. The p-value of enrichment or depletion was predicted with the fisher test, Bonferroni correction. Control group - archaea without sheath, test group - archaea with sheath.
6. Previously identified (pro)viruses of sheathed archaea collected from Medvedeva et al. 2023 and Laso-Perez et al. 2023. The predicted virus morphology, host and genome length are specified. The id of sheath-like protein is corresponding to Supplementary Data 1.
7. The IMG/VR data of predicted viruses infecting sheathed archaea. The las two columns correspond to the reference protein used for identification (SH-like or MCP) and protein identity of blastp hit.
